# Effect of Wenxia Changfu Formula Combined With Cisplatin Reversing Non-Small Cell Lung Cancer Cell Adhesion-Mediated Drug Resistance

**DOI:** 10.3389/fphar.2020.500137

**Published:** 2020-09-17

**Authors:** Meng-Ran Wang, Rui-Jie Chen, Fang Zhao, Hong-Hua Zhang, Qian-Yu Bi, Ya-Nan Zhang, Yin-Qiang Zhang, Zhi-Chun Wu, Xu-Ming Ji

**Affiliations:** ^1^ School of Basic Medical Science, Zhejiang Chinese Medical University, Hangzhou, China; ^2^ Medical College, Hangzhou Normal University, Hangzhou, China; ^3^ College of Traditional Chinese Medicine, Shandong University of Traditional Chinese Medicine, Jinan, China; ^4^ Department of Hepatic Diseases, Xiyuan Hospital, China Academy of Chinese Medical Sciences, Beijing, China

**Keywords:** Wenxia Changfu Formula, lung adenocarcinoma, cell adhesion-mediated drug resistance, integrin β1, colocalization

## Abstract

Non-small cell lung cancer (NSCLC), the major form of primary lung cancer, is a common cause of cancer-related death worldwide. Cell adhesion-mediated drug resistance (CAM-DR), a form of chemotherapy resistance, has been reported to confer resistance to various chemotherapeutic agents. Integrin β1 signaling plays an important role in CAM-DR and has been proposed as a potential target for NSCLC. Wenxia Changfu Formula (WCF) is a Traditional Chinese Compound Prescription for the intervention treatment of NSCLC combined with cisplatin (DDP). This study aims to investigate the effect and mechanism of WCF combined with DDP in reversing CAM-DR. Firstly, the chemical profile of WCF was characterized by UPLC/Q-TOF-MS analysis. A total of 237 compounds with mzCloud Best Match of greater than 70 were identified by using the online database mzCloud. Secondly, we established A549 three-dimensional(3D) cells cultured in vitro and nude mice xenografts models of the A549 cell line with Integrin β1 overexpression. *In vitro*, the cell viability, migration and adhesion were measured though MTT Assay, Wound Healing Assay and Cell Adhesion Assay, the Integrin β1 expression of the A549 cells was assessed through immunocytochemistry; *in vitro*, the transplanted tumor morphology and the colocalization of Integrin β1 and its ligands were tested by HE staining and immunofluorescence. As a result, we found that the combination effectively reduced cell viability, suppressed migration and adhesion, and downregulated the protein level of Integrin β1 in three-dimensional cultured A549 cells. And the combination of WCF with DDP significantly inhibited tumor growth, increased organelle vacuolations and decreased colocalization of Integrin β1 and its ligands including fibulin-2 and laminin. Taken together, our results confirm that the combination of WCF with DDP could reverse the lung cancer CAM-DR through the Integrin β1 signaling pathway.

## Introduction

Lung cancer is the most malignant tumor with the highest morbidity and mortality (Lee and Cheah, 2019), accounting for about 13% of total cancer cases (Zhang et al., 2020). Non-small cell lung cancer (NSCLC) accounts for about 85% of all lung cancers, and the 5-year patient survival rate of lung cancer remains low (Duma et al., 2019; Kaseda, 2020). Chemotherapy remains the primary method for NSCLC (Baltes et al., 2020). However, resistance is one of the major limitations of successful platinum treatment in NSCLC patients (Guo et al., 2020). Hence, overcoming chemoresistance is vital for the successful treatment of NSCLC.

The molecular interaction between tumor cells and microenvironment is considered to be the onset of chemotherapy resistance, namely cell adhesion mediated drug resistance (CAM-DR). CAM-DR, a form of chemoresistance, is mediated by the adhesion of tumor cells to the extracellular matrix (ECM) which enhances tumorigenicity and suppresses chemotherapy‐induced apoptosis to confer resistance to chemotherapeutic agents (Hassan et al., 2016). Integrins the heterodimeric adhesion that molecules possess, depending on their subunit composition, the capability to bind ECM components further mediating CAM-DR (Kumagai et al., 2019). Integrin β1 is almost ubiquitously expressed in cancer cells, which closely related to CAM-DR. Many studies have demonstrated that the Integrin β1 plays a crucial role in breast cancer, colorectal cancer and melanoma, because cells will be resistant to cytostatic drugs when binding to ECM components such as fibulin-2, collagen, and laminin (Yang C. et al., 2020; Yang X. et al., 2020). Integrin β1 appears as a relevant candidate for mediating CAM-DR (Jakubzig et al., 2018; Wantoch von Rekowski et al., 2019). Therefore, inhibition of Integrin β1 expression may be a potential target to overcome chemoresistance.

Traditional Chinese Medicine (TCM) has been shown to have an inhibitory effect on several cancers (Xiang et al., 2019). Our previous study proved that the WCF combined with different doses of DDP can inhibit the proliferation of tumor cells. We drew a conclusion that the combination produced a synergism in NSCLC (Ji et al., 2011; Ji et al., 2016). However, the exact mechanism of reversing drug resistance by the WCF combined with DDP remains poorly understood.

CAM-DR plays an important role in drug resistance through the adhesion between Integrin β1 and ECM. We hypothesized that the reversal of drug resistance is related to the decline of ECM proteins or participators of CAM-DR.

In the present study, we examined the effect of WCF combined with DDP on A549 cells cultured in the 3D matrix. We assessed cell viability, migration, and adhesion, tumor inhibition, expression of Integrin β1. At the same time, we established nude mice xenografts models with Integrin β1-overexpressed A549 cells, then detected transplanted tumor morphology, and analyzed colocalization of Integrin β1 and ligand proteins.

## Materials and Methods

### Drug Materials and Sample Preparation for UHPLC/MS Analysis

The WCF is composed of *Aconitum carmichaeli Debeaux* (12g), *Rheum palmatum L.* (12g), *Panax ginseng C.A.Mey* (9g) and *Angelica sinensis* (*Oliv.*) *Diels* (6g), as a fixed ratio of 4:4:3:2. These crude drugs were bought from the Shandong ZhongLu hospital (Jinan, China) and authenticated by Prof. F. Li. Detailed information on the drug materials and the scan of the vouchers were given in **Supplementary Table 1**. The mixture of *A. carmichaeli Debeaux* and *P. ginseng C.A.Mey* was macerated for 1 h and decocted for 2 h, *A. sinensis* (*Oliv.*) *Diels* was added and decocted for 30 min, and then *R. palmatum L.* was added and decocted twice for 15 min each. The filtrates were blended and concentrated to 2 g crude drug/mL, which was stored at 4°C and filtered through a 0.22 μm membrane before using.

### UHPLC/MS Analysis

UHPLC/MS analysis was performed on Thermo Fisher^TM^ UltiMate 3000 RS system coupled to a Thermo Scientific^TM^ Q Exactive high-resolution mass spectrometer (Thermo Fisher Scientific, San Diego, CA). For UPLC separation, 2 μL of sample solution was injected into a Thermo Hypersil GOLD column (100×2.1 mm, 1.9 μm). The mobile phase consisted of MeCN containing 0.1% (v/v) formic acid (A) and water containing 0.1% (v/v) formic acid(B). Linear gradient elution was applied (0–5 min, 2–20%A; 5–10 min, 20–50%A;10–25 min, 50–95%A;26–30 min, 2%A) at a flow rate of 0.3 mL/min. The column temperature was 35°C. For MS detection, the accurate mass was maintained in full scan/data-dependent MS2 (full MS/dd-MS2) mode. The operating parameters in negative ion mode were as follows: spary voltage, 3.8 kV; sheath gas pressure, 40 arb; Aux gas pressure, 10 arb capillary temperature, 300°C. MS data were acquired and processed by CD 2.1 software (Thermo Fisher), and contrasted by using the online database mzCloud (mzCloud, mzVault, ChemSpider).

### WCF-Containing Serum Preparation

Ten Wistar-Kyoto rats (5 male and 5 female, 12-weeks of age, weight 200 ± 20 g) were obtained from the laboratory animal center of the Shandong University of Traditional Chinese Medicine (Jinan, China). On the basis of preliminary studies, the rats were randomly divided into a medicated serum group and a normal serum group. The rats of the medicated serum group were given WCF (0.35 g/kg) by gavage, while rats of the normal group were given the same volume of saline 2 times per day for 3 days. The rats were starved for 12 h after given the last administration of WCF with a one-day dosage. Blood was drawn after 1 h, inactivated at 56°C for 30 min, freeze-dried, and stored at −70°C. Initial batch to batch consistency studies, performed using high-performance liquid chromatography (HPLC), have been reported in our previous paper.

### Cell Culture

The NSCLC cell lines- A549 and H1299 and the human bronchial epithelial (HBE) cells were purchased from the Cell Bank, Type Culture Collection, Chinese Academy of Sciences (Shanghai, China) and cultured in Roswell Park Memorial Institute (RPMI)-1640 medium supplemented with 10% FBS (Gibco, Beijing, China). All cell lines were cultured in a humidified incubator with 5% CO_2_ at 37°C. When the cells reached 80–90% confluence, they were passaged using 0.25% trypsin and suspended to 5.5 × 10^6^ cells/mL concentration for the experiments.

Agarose (2g; Sigma Aldrich, St Louis, MO, USA) was dissolved in 100 mL deionized water, disinfected for 30 min, and 0.5mL of the solution was added per well into a 24-wells plate after it cooled down to 50–60°C. Then, 0.5 mL of the A549 cell suspension was added per well into a 24-well plate precoated with agarose. After shaking 10 times gently, the cells were cultured for 48 h in a humidified incubator with 5% CO_2_ at 37°C.

### MTT Assay

Cell viability of H1299, HBE and A549 in normal two-dimensional (2D) culture condition and on three-dimensional (3D) materials were determined by MTT assay. The 2D cells and 3D cells were seeded at 1×10^6^ cells/well into 96-well plates and given the following treatments for 12, 24, or 48h: control, normal serum with DDP (10 μg/mL), various concentrations WCF-containing serum (5, 10, and 20%) with DDP (10 μg/mL) and 20% WCF-containing serum. Each group was set 3 wells. Absorbance in each well was measured at 570nm by ELISA reader (SpectraMax M3, Molecular Devices, USA). Calculating the viability rate uses the following formula: viability rate (%) =(OD_sample_−OD_blank_(OD_control_−OD_blank_) ×100.

### Cell Adhesion Assay

The 3D cells, divided into three groups, were given the following treatments for 24 h: control, normal serum with DDP (10 μg/mL), and 10% WCF-containing serum with DDP (10 μg/mL). Then several cell clusters were drawn out and resuspended in PBS, centrifuged for 5 min at 1,000 r/min, resuspended in RPMI-1640 containing 0.1% bovine serum albumin (BSA) at a final concentration of 5 × 10^5^ cells/mL. The RPMI-1640 containing 0.1% BSA was used as the negative control. The 0.1 mL cell suspension was added into three wells of a 96-well plate coated with 10.6 mg/mL Matrigel (Gibco, USA). The cells were incubated for 2 h at 37°C in 5% CO_2._ After incubation, the cells that had not adhered to the Matrigel were removed with phosphate-buffered saline (PBS). Then, 50 μL 0.5% crystal violet solution was added per well, incubated for 5 min, and washed with PBS. The cells were cultured overnight at 37°C in the dark. After 24 h, 150 µL methanol was added per well, and the absorbance was measured at a wavelength of 630 nm. The adhesion inhibition rate was calculated according to the following formula: $\text{Adhesion Inhibition rate }(\%)\;=\;\frac{Matrigel-BSA}{BSA}\times 100$.

### Wound Healing Assay

Cell invasion of A549 was analyzed using wound healing assay. The A549 cell line was cultured in 3D conditions and treated as mentioned above, and then several cell clusters were drawn out and resuspended in PBS, centrifuged and resuspended in RPMI-1640. A549 cells were seeded at 5 × 10^5^ cells/well into 96-well plates and allowed to grow for about 12 h. After respectively incubated, scratch wounds were photographed immediately as 0 h. And then scratch wound images of the same field were photographed at 24 h after scratch under a phase contrast microscope (Leica, Nussloch, Germany). The measurement of the wound surface area was calculated with Image J software. The percent wound closure was calculated according to the following formula: $\text{Percent wound closure }(\%)\;=\;\frac{Area0h-Area24h}{Area24h}\times 100$.

### Immunocytochemistry

The A549 cell line was treated as above. The Integrin β1 expression of the A549 cells was assessed through immunocytochemistry using an antibody against Integrin β1 (1:500; Rabbit Polyclonal, AB179471). The slides of A549 cells were blocked using a blocking buffer for 1 h at 25 °C and stained with the specific primary antibody for 24 h. The staining of Integrin β1 expression was visualized as a brown color in the cell membrane. Image-Pro Plus 6.0 software (Media Cybernetics Inc., USA) was used for image analysis, and the positive staining integral optical density (IOD) was detected within 3 random ﬁelds at 400× magniﬁcation.

### Xenograft Studies Integrin β1 Over-Expressed in A549

The A549 cells were transfected with two kinds of eukaryotic expression plasmids, including pcDNATM3.1-GFP-Integrin β1 and pcDNATM3.1-GFP (positive control plasmid) by liposomes (Lipofectamine^TM^ 2000, Invitrogen) to test the expression efficacy of the genes according to the manufacturer’s instructions. The A549 cells were replated into 6-well plates at a density of 3 × 10^5^ cells. 2 μL of lipofectamine 2000 (Invitrogen, Life Technologies, Carlsbad, CA, USA) was incubated with 1 μg of the indicated plasmid for 30 min at 25 °C, and which added into the A549 cells, incubated at 37 °C in a humidified atmosphere of 5% CO_2_ before being harvested. The uptake of plasmid DNA was primarily evaluated by counting the number of GFP-positive cells, thus attaining an estimate of the transfection efficiency.

Female STOCK-Foxn1nu/NJu nude mice, 4 weeks of age, were purchased from the Model Animal Research Center of Nanjing University (SCXK 2015-0001) and were maintained at the Animal experimental center of Shandong University of Traditional Chinese Medicine in a specific pathogen-free environment (24 ± 2°C, 50% ± 10% humidity) with food and water provision. All experiments were conducted with the approval of the Institutional Animal Care and Use Committee and were in compliance with the National Institutes of Health Guidelines for Use and Care of Laboratory Animals.

Nude mice were implanted subcutaneously on the right subaxillary with 5×10^7^ A549 cells with Integrin β1 over-expression (0.2 mL/mouse). The model mice were randomly divided into three groups with 10 mice per group: The WCF and DDP combination group (WCF group) was administered i.p. at doses of 4 mg/kg/d DDP twice a week and administered i.g. at doses of 40 g/kg WCF once a day (**Supplementary Table 2**). The DDP group was administered i.p. at doses of 4 mg/kg DDP twice a week and i.g. with the same volume of PBS instead. The control group was injected with the same volume of PBS instead. After 28 days injection, mice were sacrificed and subcutaneous tumors were harvested, weighed and cut into two parts: one part (1 mm^3^) was fixed in 2.5% glutaraldehyde for ultrastructure observation; the another part was fixed in 10% formalin for HE staining and immunofluorescence staining. The inhibitory rate was calculated with the formula: Inhibitory rate (%) = [(1−average tumor weight in the treated group)/average tumor weight in the control group] ×100.

### Electron Microscopy

Tumor tissues of 1 mm × 1 mm × 1 mm volume were sectioned and fixed with 2.5% glutaraldehyde for 10 min immediately. They were rinsed in distilled water, dried, and embedded using 0.13% methyl cellulose and 0.4% uranyl acetate for 10 min and cut into slices(60–80nm). After drying, the samples were photographed by using a transmission electron microscope (JEM-1400, JEOL, Tokyo, Japan) at 4,000× and 5,000× magnification.

### Immunofluorescence

The 10% formalin fixed tumor tissues were embedded in paraffin and then cut at a thickness of 4–6μm. The tissue slice was stained immunofluorescence according to the kit instructions by using a streptavidin–biotin complex method after regular dewaxing and antigen retrieval. Paraffin sections were blocked by a blocking buffer for 1 h at 25 °C. Slides were incubated overnight with Integrin β1 antibody (1:200; mouse Polyclonal, AB30388) and fibulin-2 antibody (1:100; Rabbit Polyclonal, BS0809R) or laminin antibody (1:200; Rabbit Polyclonal, AB11575). Samples were treated with the secondary antibodies FITC conjugated anti-rabbit IgG(H+L) (1:500, ZF-0311-FITC), or FITC conjugated anti-mouse IgG(H+L) (1:500, ZF-0312-FITC), and anti-mouse IgG/RBITC (ZF-0313-RBITC), or anti-rabbit IgG/RBITC (ZF-0316-RBITC) for 1 h at room temperature. Slides were finally washed, mounted in VectaShield mounting medium containing 4’,6-diamidino-2-phenylindole (DAPI) (C02-04002), and observed and photographed on an Olympus IX73 fluorescent inverted microscope (IX73-DP80, Olympus Corporation, Japanese). The quantitative analysis of immunofluorescence images was performed using Image software (ImageJ, National Institutes of Health, USA). Fluorescence Intensity of Integrin β1, fibulin-2, and laminin immunoreactivity were measured in tumor tissue to evaluate the expression level of the adhesion molecule. The degree of colocalization in the images was evaluated by measuring Manders’ coefficients M1 and M2 of each set of images. For that purpose, the plugin Coloc 2 was applied to the red and green channels of the images.

### Statistical Analysis

The Statistical Package for the Social Sciences **(** *SPSS*) 22.0 was used for the statistical analysis. The data was expressed as mean ± standard deviation ($\bar{x}$ ± *s*), and one-way analysis of variance (ANONA) and *S-N-K* method were used for comparison between groups. *P*-value <0.05 is considered statistically significant.

## Results

### Chemical Profiling of WCF

UHPLC/MS analysis was employed to characterize the chemical composition of WCF. A total of 805 compounds were putatively identified by comparing against database mzCloud, of which 237 compounds had a mzCloud Best Match score greater than 70. These compounds involved different kinds of constituents, such as aminophenols, polysaccharides, saponins, anthraquinone, organic acids, and so on. Among them, Ginsenoside and ferulic acid showed obvious anti-cancer effect. Mass spectrometry analysis was performed on some compounds with mzCloud Best Match of over 70. The results were shown in **Table 1**.

**Table 1:** Characterization of some chemical constituents in WCF by UHPLC/MS Analysis.

Name	Formula	Molecular Weight	RT(min)	mzCloud Best Match
DL-Tryptophan	C11 H12 N2 O2	204.08959	6.396	99.4
Adenosine	C10 H13 N5 O4	267.09622	5.033	98.5
Erucamide	C22 H43 N O	320.30654	25.6	95.9
Catechin	C15 H14 O6	290.07857	6.646	95.7
Adenine	C5 H5 N5	118.02801	2.239	94.5
Dibutyl phthalate	C16 H22 O4	278.1513	17.962	94.2
Ferulic acid	C10 H10 O4	194.05769	7.735	93.1
1,6-Bis-O-(3,4,5-trihydroxybenzoyl) hexopyranose	C20 H20 O14	501.11129	6.872	92.7
4-Coumaric acid	C9 H8 O3	164.04721	7.43	91.7
Nicotinic acid	C6 H5 N O2	123.03229	2.26	91.5
Emodin	C15 H10 O5	270.05252	10.909	89.7
Aloe-emodin	C15 H10 O5	270.05315	7.729	88.6
Ginsenoside Rg3	C42 H72 O13	766.4849	14.334	88.1
Linoleic acid	C18 H32 O2	280.23986	21.075	85.8
Aconitine	C34 H47 N O11	645.31433	11.902	85.7
Salsolinol	C10 H13 N O2	179.09432	3.435	85.5
Cytarabine	C9 H13 N3 O5	243.08539	2.154	85.5
Ginsenoside Rb1	C54 H92 O23	1130.585	11.173	84.5
Kaempferol-7-O-glucoside	C21 H20 O11	448.10029	9.368	84.5
Formononetin	C16 H12 O4	268.07305	10.503	82.3
Sedanolide	C12 H18 O2	194.13054	12.095	81.8
Citric acid	C6 H8 O7	192.02603	1.611	80.8
2-Hydroxycinnamic acid	C9 H8 O3	146.03661	7.448	79.4
Ginsenoside Rg2	C42 H72 O13	806.47713	14.329	77.9
Gallic acid	C7 H6 O5	170.02027	4.793	75.5
Isorhapontigenin	C15 H14 O4	258.089	9.076	72.5

### WCF Combined With DDP Inhibited Proliferation of A549 Cells Cultured in 3D Conditions

**Figure 1:**
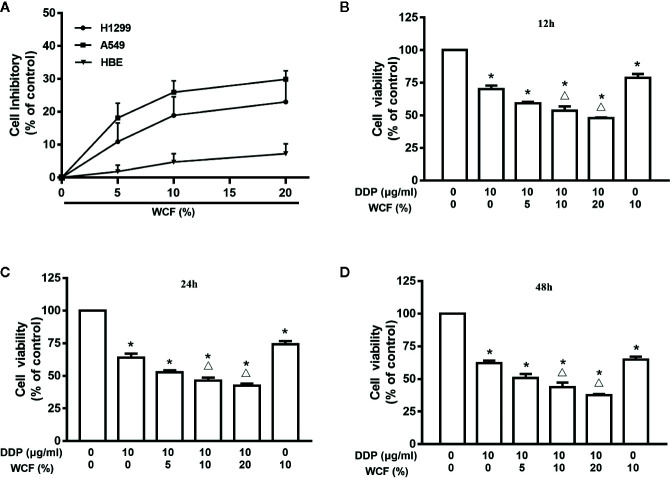
WCF reduces viability in A549 cells. **(A)** Effects of WCF on cell viability in HBE, A549 and H1299 cells. Cells were treated with various concentrations of WCF-containing serum for 24h, respectively. **(B–D)** A549 cells in 3D conditions were treated with DDP, 10% WCF-containing serum or were cotreated with DDP (10μg/mL) plus various concentrations of WCF-containing serum (5%, 10%, 20%) for 12, 24 or 48h before analysis with the MTT assay. The data were shown as the means±SD of three independent experiments. *P < 0.05 compared to control, ^△^ *P* < 0.05 compared to DDP.

We evaluated the cytotoxic activity of WCF in three cell types. Two lung cancer (A549 and H1299) and one normal human cell lines (HBE) were used in this study. Cytotoxicity was measured by incubating the cells with serum containing various concentrations of WCF for 24 h followed by MTT. Dose-dependent cytotoxicity effect curves were shown in **Figure 1A**. These results demonstrated that WCF has a significant cytotoxic activity in the lung cancer cell lines and a dramatically reduced effect on the normal cells. MTT assay was used to test the growth inhibition rates of WCF combined with DDP on A549 cells cultured in 3D conditions (**Supplementary Figure 3**). The A549 cells cultured in 3D conditions were treated with DDP (10 μg/mL) combined with various concentrations of WCF-containing serum (5, 10, and 20%). After 12, 24, and 48h of incubation, cells viability was tested and shown in **Figures 1B–D**, **Supplementary Figures 1, 2**. From the results, WCF combined with DDP suppressed cell viability on A549 cells in time and dose-dependent manners.

### WCF Combined With DDP Inhibited Cell Adhesion and Migration of A549 Cells

**Figure 2:**
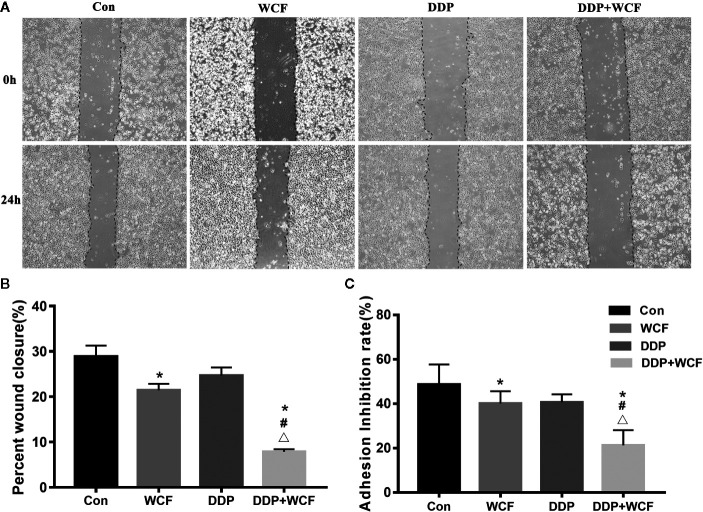
WCF combined with DDP inhibited cell migration and adhesion of A549 cells. **(A)** A549 cells were performed with wound healing assay, after which, the cells were treated with DDP and WCF-containing serum separately or in combination and photomicrographed at 0 and 24 h. **(B)** The relative wound surface area was calculated by Image J. **(C)** The adhesion inhibition rate in different groups. Statistical difference was analyzed by ANOVA, *P < 0.05 compared to control, ^#^P < 0.05 compared to WCF, ^△^P < 0.05 compared to DDP.

To explore the effects of WCF on A549 cell migratory abilities wound healing assay was performed. Because A549 cell proliferation was inhibited when the concentration of WCF was more than 10%, and the difference was not statistically significant between 10 and 20% WCF-containing serum, the concentration 10% WCF-containing serum was picked out for wound healing assay and other subsequent experiments. Photomicrographs showed that the wounds of control group cells were significantly healed after 24 h, whereas the wound healing of those receiving DDP and WCF treatment cells were inhibited (**Figure 2A**). Wound surface area analyses showed that WCF combined with DDP restrained the wound healing (**Figure 2B**).

Matrigel was a gelatinous protein mixture and resembled the complex extracellular environment in many tissues. The number of cells that adhere to Matrigel reflected their adhesive ability. As shown in **Figure 2C**, the adhesion rate of the WCF combined with the DDP group was lower than that of the WCF group, DDP group and blank control.

### WCF Combined With DDP Inhibited the Expression of Integrin β1 in A549 Cells

**Figure 3:**
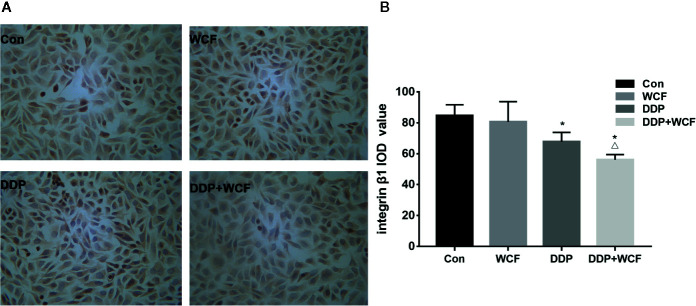
WCF Combined with DDP Inhibited the Expression of Integrin β1 in A549 Cells. **(A)** Integrin β1 immunocytochemistry of A549 cells in Control, DDP, WCF, and the combination groups. Immunocytochemistry staining images were viewed at a magnification of 400. **(B)** The integrated optical density was calculated by Image-Pro Plus 6.0 software. Statistical difference was analyzed by ANOVA, *P < 0.05 compared to control, ^△^ *P* < 0.05 compared to DDP.

Immunocytochemistry was employed to test the expression of Integrin β1 of A549 cells. A549 cells of control, DDP, WCF, and WCF combined with DDP groups were analyzed. As shown in **Figure 3**, Integrin β1 was mainly distributed on A549 cell membranes. The Integrin β1 protein expressions in DDP and WCF treatment groups were decreased compared with control group. WCF combined with DDP group significantly reduced Integrin β1 expression compared to DDP group. It was shown that WCF combined with DDP could inhibit the expression of Integrin β1.

### WCF Combined With DDP Inhibited Tumor Growth of Integrin β1 Over-Expressed Xenograft Mouse Model

**Figure 4:**
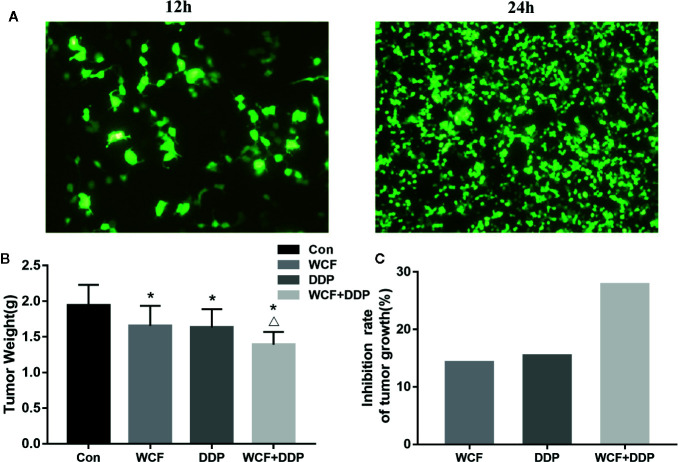
WCF combined DDP inhibited tumor growth and reduced tumor weight. **(A)** T24 and T48 Fluorescence expression in A549 cells transfected with Integrin β1 under a fluorescence microscope. **(B)** Statistical analyses demonstrated the tumor weights. **(C)** The inhibition rate of tumor growth. The data were shown as the means ± SD of ten mice. *P < 0.05 compared to control, ^△^P < 0.05 compared to DDP.

The anticancer effect of WCF combined with DDP was assessed in tumor xenograft mouse model of Integrin β1-overexpressed A549 cells (**Figure 4A**). Compared with the control group, the WCF combined with DDP-treated mice showed a significant growth-inhibitory effect. After 4 weeks of drug intervention, the tumor weight of the DDP and WCF combined with DDP groups decreased markedly compared to the control group (**Figure 4B**). The inhibition rate of tumor growth in DDP treatment was 15.97%, WCF treatment was 14.73%, while that of WCF combined with DDP group was 28.35% (**Figure 4C**). Thus, the WCF combined with DDP treatment could inhibit tumor growth and reduce tumor weight dramatically.

### WCF Combined With DDP Affected Transplanted Tumor Morphology

**Figure 5:**
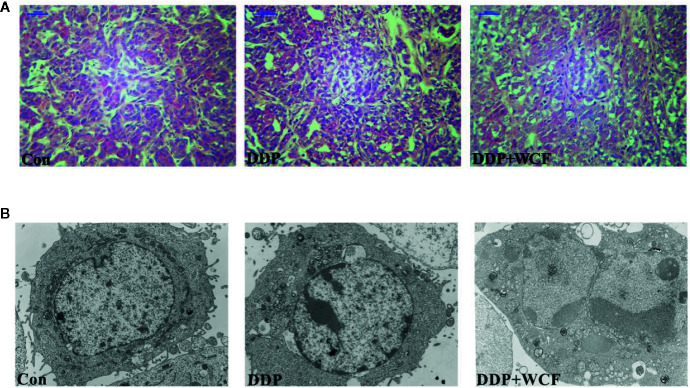
WCF combined DDP affected transplanted tumor structure and ultrastructure. **(A)** HE staining of section slices of tumor tissue in control, DDP and WCF combined DDP groups, respectively. HE staining images were viewed at a magnification of 400. **(B)** TEM of cross section slices of tumor tissue in control, DDP and WCF combined DDP groups, respectively. TEM staining images were viewed at a magnification of 5000.

The changes in microscopic structure and ultrastructure of the transplanted tumor were observed by light and electron microscopies. As shown in **Figure 5A**, HE staining demonstrated that tumor cells of the control group arranged closely and infiltrated into the surrounding tissue, where more microvascular structures were formed. Meanwhile, the tumor cells of the DDP group arranged loosely compared to the control group, a small number of necrotic foci and more microvascular in cell mesenchyme were found. Even less microvascular structures were found in cell mesenchyme. Ultrastructural observation showed that there were fewer microvilli and organelles in the DDP and WCF combined with DDP groups than the control group, and more organelle vacuolations were observed in the WCF combined with DDP group than the DDP group. Furthermore, concentrated and shrunken nucleus chromatins were observed in the DDP and WCF combined with DDP groups; increased electron density was also observed under the nuclear envelope (**Figure 5B**).

### WCF Combined With DDP Inhibited Colocalization of Integrin β1 and Its Ligand Proteins in Tumor Tissue

**Figure 6:**
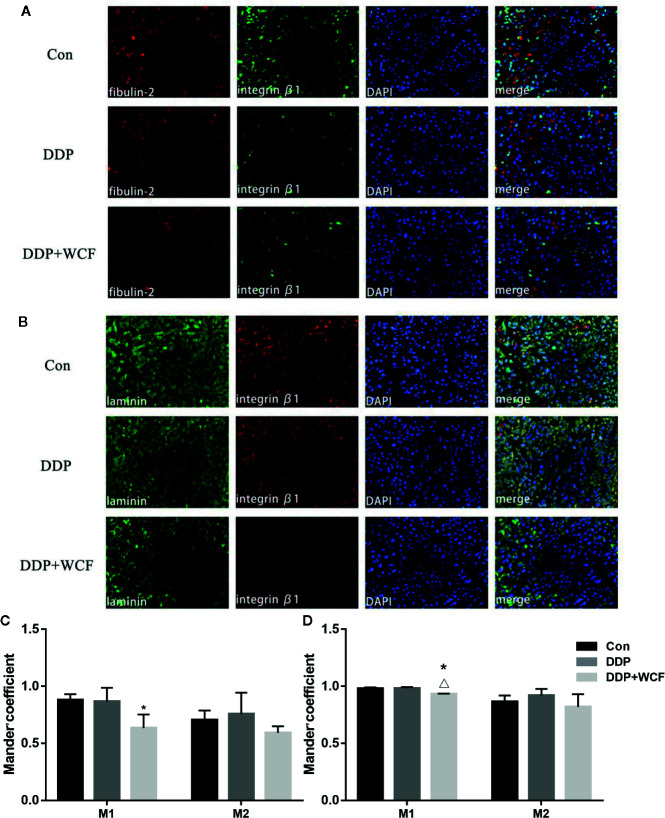
WCF combined with DDP affected expression and colocalization of Integrin β1and its ligand proteins(fibulin-2/laminin). **(A)** Immunofluorescence labeling of fibulin-2 and Integrin β1 in tumor tissue slices. **(B)** Immunofluorescence labeling of laminin and Integrin β1 in tumor tissue slices. **(C)** Mean fluorescence intensity as well as quantitative colocalization analysis of fibulin-2 and Integrin β1. **(D)** Mean fluorescence intensity as well as quantitative colocalization analysis of laminin and Integrin β1. Mean fluorescence intensity and Manders’coefficient were calculated by Image J. Statistical difference was analyzed by ANOVA, *P < 0.05 compared to control, ^△^ *P* < 00.05 compared to DDP.

The colocalization of Integrin β1 and its ligands were tested by immunofluorescence. Our observations of tumor slices revealed that Integrin β1 and its ligands, such as fibulin-2 and laminin staining could be clearly viewed in all the groups. As shown in **Figure 6**, **Supplementary Figure 4**, the Integrin β1 staining was found in the cell membrane, fibulin-2 positive staining in the ECM, and laminin positive staining was located in the cytoplasm and the ECM.

Quantitative colocalization analysis of digital immunofluorescence images further demonstrated the overlap of Integrin β1 and its ligand staining in tumor tissue. Here, full images in each of the groups were evaluated using the Manders’ colocalization coefficients. The average M1 coefficient of fibulin-2 and Integrin β1 in the control group was 0.88, indicating that approximately 88% of the fibulin-2 staining in the entire image colocalized with the imaged Integrin β1; the average M2 coefficient (i.e., the portion of Integrin β1 that colocalized with the imaged fibulin-2) was 0.70. In the DDP group and WCF combined group, the M1 and M2 coefficients of fibulin-2 and Integrin β1 were 0.87 and 0.76, 0.63 and 0.59, respectively. According to statistical analysis, M1 coefficients in the WCF combined with the DDP group were significantly lower than those in the control group. The average M1 coefficient of laminin and Integrin β1 in the control group was 0.98, indicating that approximately 98% of the laminin staining in the entire image colocalized with the imaged Integrin β1; the average M2 coefficient (i.e., the portion of Integrin β1 that colocalized with the imaged laminin) was 0.86. On the other hand, the M1 and M2 coefficients of laminin and Integrin β1 in the DDP group and WCF combined group were 0.98 and 0.92, 0.93 and 0.82, respectively. M1 coefficients in the WCF combined with the DDP group were significantly lower than those in the control group and DDP group (**Figures 6C, D**).

## Discussion

NSCLC is epithelium-derived lung cancer including adenocarcinoma and squamous carcinomas and large cell carcinoma, accounting for nearly 80% of all newly diagnosed lung cancer cases (Wald, 2018). The first-line chemotherapeutic agents for the treatment of human NSCLC is chemotherapy based on platinum drugs (Quarta et al., 2019). However, the usage of platinum drugs is constricted partly due to the drug resistance and toxic side effects. Thus, the discovery of effective clinical therapies for reducing the drug noxious side effects and reversing drug resistance of NSCLC patients is deemed necessary.

The tumor microenvironment (TME) has been regarded as a pivotal player in the development of chemoresistance (Da Ros et al., 2018; Russi et al., 2019; Wang et al., 2020). There is a mutual stimulation between the components of TME and the tumor cells, thereby promoting tumor cell adhesion, migration, and invasion (Wang et al., 2017). The ECM of TME acts as a key barrier to the diffusion of chemotherapeutic drugs by forming thick fibers within the tumor, and the remodeling of fibers promotes the drug resistance of tumor cells. The Integrins are an important cell surface molecule that mediates a variety of biological functions, including adhesion between cells and the ECM. Thus, targeting the ECM, Integrins and the interaction between them in CAM-DR might be an effective method to control cancer chemoresistance.

Based on different body conditions of patients with cancer, TCM uses different treatments to intervene TME so that the microenvironment of the body can reach a “steady state”, thereby inhibiting the occurrence and metastasis of the tumor (Liu et al., 2015). The WCF is composed of *A. carmichaeli Debeaux*, *R. palmatum L.*, *P. ginseng C.A.Mey* and *A. sinensis* (*Oliv.*) *Diels*, and the combination of the four herbs provides insights into the pathogenesis of lung cancer from the perspective of traditional Chinese medicine. Studies have shown that the active ingredients of the WCF components such as aconitine, ginsenoside Rh2 (Li et al., 2020), ginseng peptide GS9, Rg3 (Wang et al., 2018), emodin, Angelica polysaccharide (Zhang et al., 2017) and Angelica volatile oil have anti-tumor activities and can reverse the resistance of the tumor to drugs. Furthermore, the chemical profile of WCF was characterized for the first time and a total of 26 compounds were identified by UPLC/MS analysis.

Previous studies have indicated that the combination of WCF and DDP have a signiﬁcant inhibitive effect on tumor proliferation both *in vitro* and *in vivo* (Ji et al., 2011; Ji et al., 2016). However, the A549 cells were cultured in a normal plate in our previous study, which did not reflect the real environment of the tumor cells, which needed to be considered in further studies.

The 3D culture technology can be used to simulate the TME since the tumor environmental factors play a key role in drug resistance (Virumbrales-Muñoz et al., 2019). Studies have shown that multicellular 3D culture and interaction with stromal components are considered to be the basic elements for establishing a ‘more clinically relevant’ tumor model. It is widely used in A549 cells, MDA-MB-231 cells, melanoma cell line SK-Mel-28 cells, and prostate epithelial tumor promoter cells (Fetah et al., 2019). Agarose has been widely used as a scaffold for cell culture and has also been successfully used in the 3D culture of tumor cells (Kletzmayr et al., 2020). In 3D model, the architecture of cancer tumor is more complex, cell spheroids are commonly divided into 3 layers: the outer layer with high proliferation rates, the middle layer with senescent cells, and the hypoxic core with necrotic cells (Amann et al., 2014; Hoarau-Véchot et al., 2018). The level of oxygen and nutrients is lower in the core due to the increase of cell density and integrin β1 binding extracellular matrix, the oxygen and nutrition levels in the core region are low, which reflects the situation observed in solid tumors, and the volume of solid tumors can reach 60% under chronic hypoxia (Gomes et al., 2019). Persistent hypoxia in the core leads to glycolysis and increased synthesis of pyruvate, lactic acid and carbon dioxide, which acidifies the medium (Pouysségur et al., 2006). Cells have to adapt to this harsh environment to survive. Therefore, some cytotoxic agents such as 5-FU, doxorubicin, and cisplatin, which work with oxygen, are poor effective in 3D models (Li et al., 2010). We have already established A549 cells on 3D cell culture system that were incubated with DDP alone or in combination with WCF medicated serum *in vitro*. With the extension of culture time, when cells formed multicellular colonies, the tolerance of cells to DDP increased, and the sensitivity of cells to DDP decreased (Zhang et al., 2019). In this study, the WCF combined with DDP not only suppressed cell viability on A549 cells and H1299 cells in time and dose-dependent manners but also significantly inhibited the adhesion and migration abilities of tumor cells. Simultaneously, it had no effect on HBE *in vivo*.

Integrin β1 has been reported to be up-regulated in non-small cell lung cancer, breast cancer, ovarian cancer, liver cancer, bladder cancer and melanoma. Its expression is positively correlated with tumor cell viability, adhesion and migration. At present numerous studies have pointed out that Integrin β1 has been reported to be one of the most significant adhesion molecules that has been suggested to be involved in CAM-DR (Xu et al., 2017). The tumor cells appear a resistant state against cytostatic drugs when Integrin β1 binds to ECM components (Iyoda et al., 2016). We made use of the 3D cell model, and established nude mice xenografts models with Integrin β1-overexpressed A549 cells. The results highlighted that DDP decreased the expression of Integrin β1, while the combination of WCF with DDP further reduced the expression of Integrin β1 *in vitro*.

We also studied the anti-tumor effect and transplanted tumor morphology of this combination with animal experiments. WCF combined with DDP could inhibit tumor growth dramatically, enlarge the intracellular space and concentrate and shrunk nucleus chromatins. Our results indicated that the combination was related to the inhibition of tumor growth, increased cell apoptosis, decreased cell adhesion and enlarged intracellular space of tumor cells. Based on these literatures and our results, we speculate that deletion of Integrin β1 makes it easier for drugs to enter the body, increases intracellular drug concentration, and improves the sensitivity of tumor cells to drugs.

Recently, increasing evidence has indicated that the high expression of Integrin β1 can enhance the adhesion of tumor cells to the EMC, and the binding of Integrin β1 with adhesion molecules in ECM can lead to tumor cells resistance to chemotherapy drugs by inhibiting cell apoptosis (Shen et al., 2019). Fibulin-2 is the main ECM protein and interacts with surface receptors such as cell-matrix proteins and Integrins to play an important role in cell adsorption and migration (Fontanil et al., 2014). Meanwhile, laminin is known to affect cellular processes, such as differentiation, adhesion, and migration through laminin–Integrin interactions. Focal adhesives formed by ECM–Integrin–cytoskeletal proteins are the structural basis of Integrin signal transduction. Basically, many signaling proteins play a role in Integrin-mediated signal transduction by binding to focal adhesives. The extracellular region of Integrin is connected with the ECM, while the intracellular region is connected with the cytoskeleton to form focal adhesion (FAP), which mediates the adhesion of cells to the ECM and the bidirectional transmission of intracellular and extracellular signals (Khadilkar et al., 2020). Since those proteins and their interactions are involved in cancer growth and metastasis, there is a need to develop drugs targeting Integrins, ECM components and/or the interactions in the tumors.

To further study the mechanism by which the combination of WCF and DDP reverses CAM-DR through the Integrin β1 pathway tested the colocalization of Integrin β1 and its ligand proteins by immunofluorescence. The colocalization of Integrin β1 and its ligand such as fibulin-2, laminin are inhibited by WCF combined with DDP in our nude mice model. It suggests that the expression and binding of ECM and its receptors are also involved in decline.

Although there are important discoveries in this study, some limitations are also there, such as the interaction between the Integrin β1 and its ligands, and the exact mechanism involved in drug resistance decline. Therefore, further studies need to be carried out in the future.

## Conclusion

In summary, WCF combined with DDP inhibited the cell proliferation, adhesion and migratory of A549 cells and inhibited Integrin β1 expression dramatically in 3D conditions

In addition, WCF combined with DDP inhibited the tumor growth remarkably in our nude mice models, and the expression of colocalization Integrin β1 and its ligands was inhibited. Altogether, we can draw a preliminary conclusion that WCF combined with DDP reverses the CAM-DR, perhaps by inhibiting the expression of Integrin β1 and its ligand proteins.

## Data Availability Statement

All datasets generated for this study are included in the article/**Supplementary Material**.

## Ethics Statement

All animal experimental procedures were reviewed and approved by the “Animal Welfare and Ethics Committee of Shandong University of Traditional Chinese Medicine for Nationalities”, and animal care was conducted in accordance with institutional guidelines.

## Author Contributions

X-MJ and Z-CW are designed the study and drafted and finalized the manuscript. M-RW, R-JC, and FZ made important suggestions regarding the experimental procedure, along with critically reviewing the paper. H-HZ carried out the vitro research. Q-YB conducted animal experiments. Y-NZ, M-RW, and Y-QZ carried out statistical analysis. All authors contributed to the article and approved the submitted version.

## Funding

This research was supported by the National Natural Science Foundation of China (No.81774198, 81703839, 81573871, 81273634), the Open Project of Shandong Co-Innovation Center of Classic TCM Formula (NO:2018KFZ03), and National Key R&D Program of China under Grant (No. 2019YFC1708700, 2019YFC1708702), the Postgraduate Scientific Research Fund of Zhejiang Chinese Medical University.

## Conflict of Interest

The authors declare that the research was conducted in the absence of any commercial or financial relationships that could be construed as a potential conflict of interest.
