# Supplementary figures and images for: Effect of Wenxia Changfu Formula Combined With Cisplatin Reversing Non-Small Cell Lung Cancer Cell Adhesion-Mediated Drug Resistance
Source: Front Pharmacol. 2020 Sep 17;11:500137. doi: 10.3389/fphar.2020.500137 (PMC7527591; doi:10.3389/fphar.2020.500137)

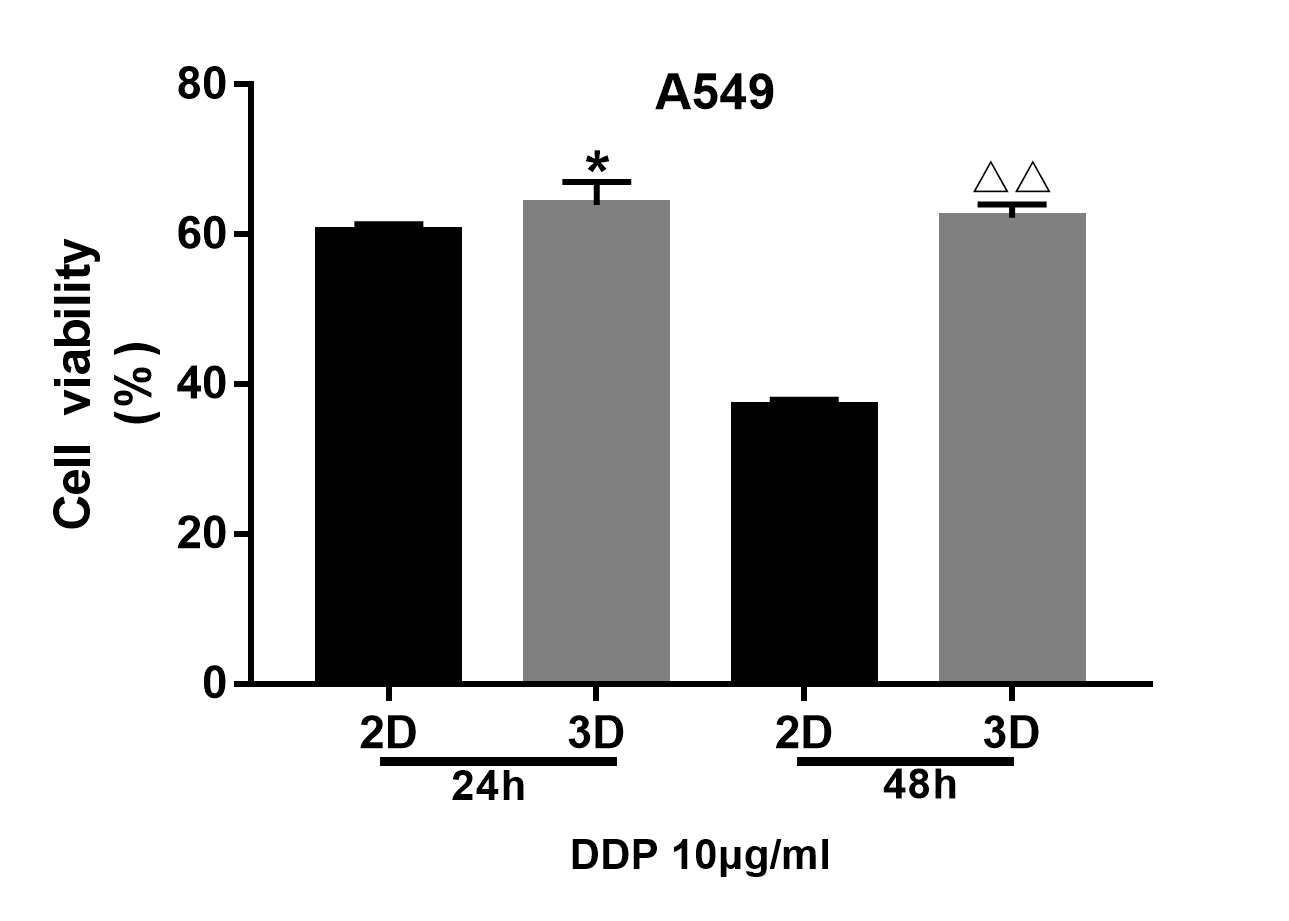

Supplement: Supplementary file 1 [file Image_1.png]

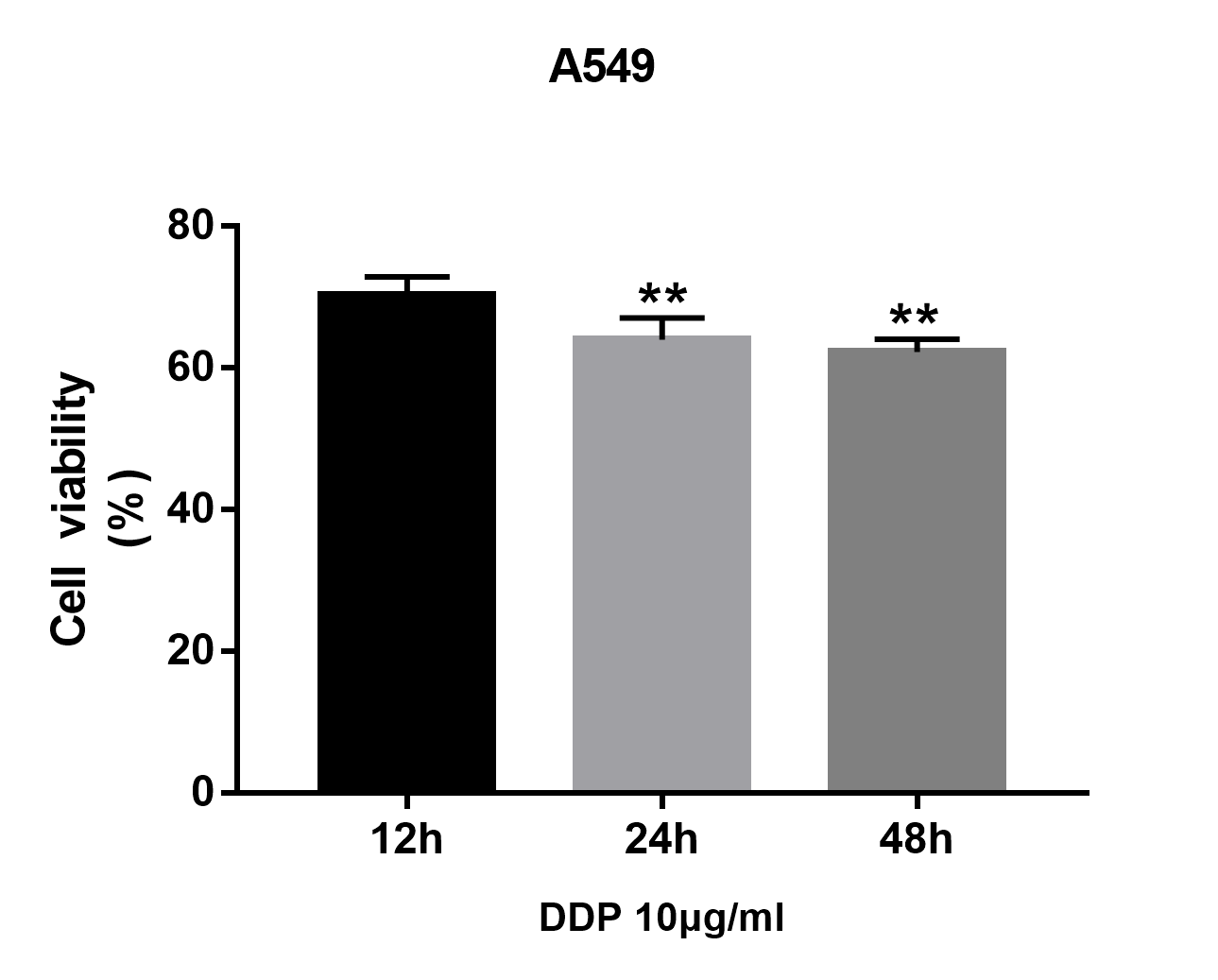

Supplement: Supplementary file 2 [file Image_2.png]

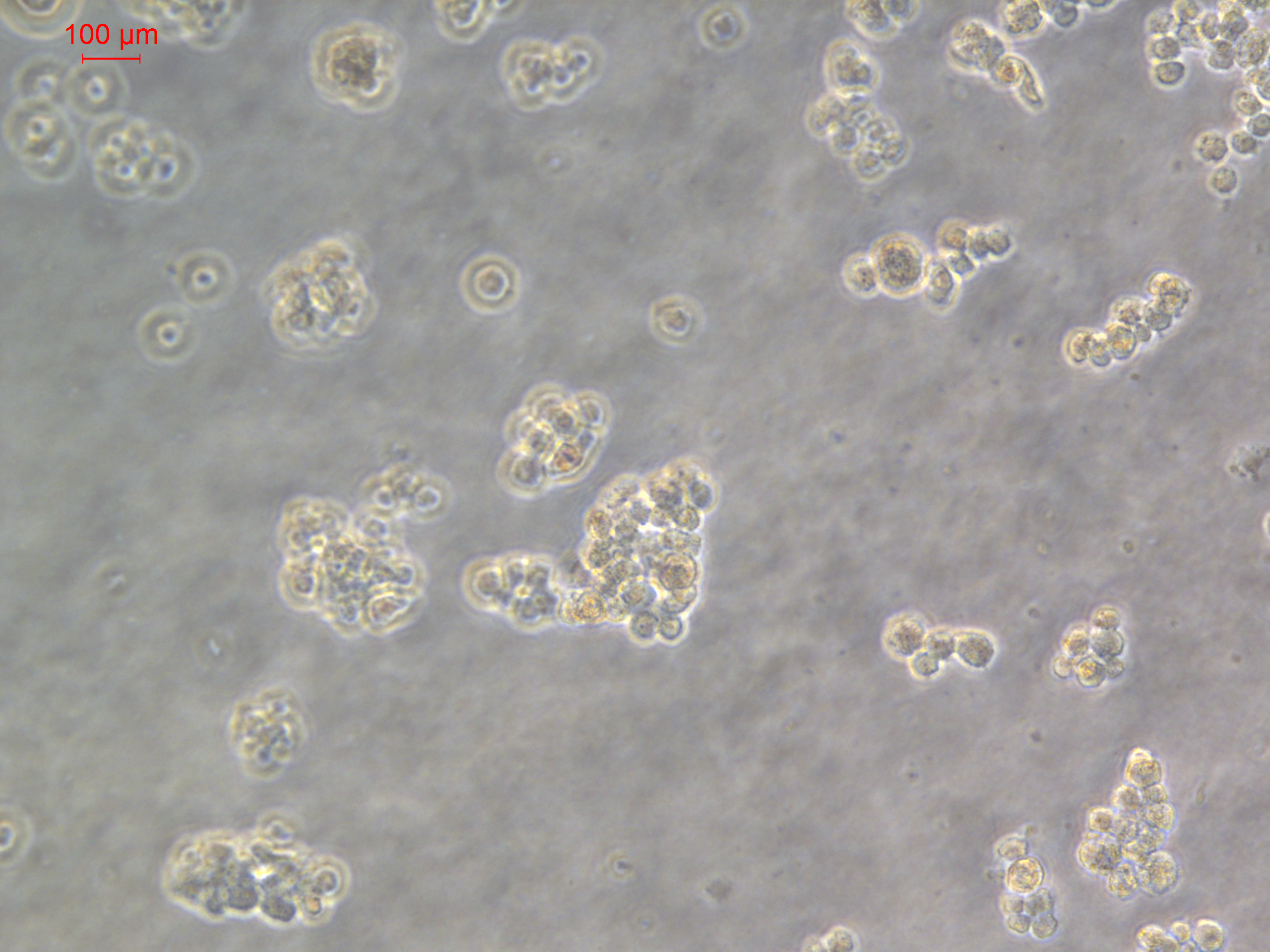

Supplement: Supplementary file 3 [file Image_3.png]

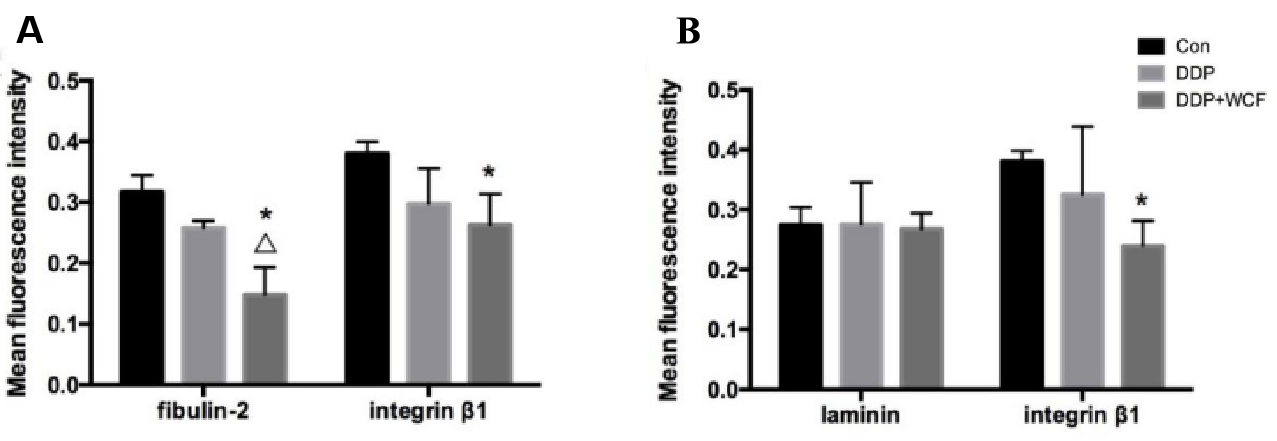

Supplement: Supplementary file 4 [file Image_4.png]
